# Axonal Transmission in the Retina Introduces a Small Dispersion of Relative Timing in the Ganglion Cell Population Response

**DOI:** 10.1371/journal.pone.0020810

**Published:** 2011-06-02

**Authors:** Günther Zeck, Armin Lambacher, Peter Fromherz

**Affiliations:** 1 Department of Systems and Computational Neuroscience, Max Planck Institute of Neurobiology, Martinsried, Germany; 2 Neurochip Research Group, Natural and Medical Sciences Institute at the University of Tübingen, Reutlingen, Germany; 3 Department of Membrane and Neurophysics, Max Planck Institute for Biochemistry, Martinsried, Germany; Dalhousie University, Canada

## Abstract

**Background:**

Visual stimuli elicit action potentials in tens of different retinal ganglion cells. Each ganglion cell type responds with a different latency to a given stimulus, thus transforming the high-dimensional input into a temporal neural code. The timing of the first spikes between different retinal projection neurons cells may further change along axonal transmission. The purpose of this study is to investigate if intraretinal conduction velocity leads to a synchronization or dispersion of the population signal leaving the eye.

**Methodology/Principal Findings:**

We ‘imaged’ the initiation and transmission of light-evoked action potentials along individual axons in the rabbit retina at micron-scale resolution using a high-density multi-transistor array. We measured unimodal conduction velocity distributions (1.3±0.3 m/sec, mean ± SD) for axonal populations at all retinal eccentricities with the exception of the central part that contains myelinated axons. The velocity variance within each piece of retina is caused by ganglion cell types that show narrower and slightly different average velocity tuning. Ganglion cells of the same type respond with similar latency to spatially homogenous stimuli and conduct with similar velocity. For ganglion cells of different type intraretinal conduction velocity and response latency to flashed stimuli are negatively correlated, indicating that differences in first spike timing increase (up to 10 msec). Similarly, the analysis of pair-wise correlated activity in response to white-noise stimuli reveals that conduction velocity and response latency are negatively correlated.

**Conclusion/Significance:**

Intraretinal conduction does not change the relative spike timing between ganglion cells of the same type but increases spike timing differences among ganglion cells of different type. The fastest retinal ganglion cells therefore act as indicators of new stimuli for postsynaptic neurons. The intraretinal dispersion of the population activity will not be compensated by variability in extraretinal conduction times, estimated from data in the literature.

## Introduction

Visual information is transmitted from the eye to the brain in trains of action potentials originating from populations of projection neurons, the retinal ganglion cells. A single visual stimulus excites more than a dozen different types of ganglion cells each of them tiling the retinal surface in a mosaic-like fashion [1], [2], [3], [4]. Each ganglion cell type may respond with a different latency to the same stimulus because of its presynaptic circuitry that is shaped by specific types of interneurons [3], [4].

If the brain uses the information of different response latencies it requires the knowledge about stimulus onset. A recent study in the auditory system proposed that threshold crossing of summed spiking activity may signal the occurrence of a new stimulus [5]. Alternatively, the first spikes in the population response may act as a ‘visual switch’ [3] providing an internal reference for stimulus onset. Spike latencies referenced to stimulus onset carry information additional to that encoded by the spike rate as demonstrated in different sensory modalities, including visual [6], [7], [8], [9], somatosensory [10], [11] and auditory systems [5], [12]. Furthermore, time-lagged correlations between retinal ganglion cells facilitate rapid stimulus encoding [13], [14] that may be used by animals in stimulus discrimination tasks [7]. These studies exemplify the impact of spike timing in a neuronal population. However, latencies or relative time differences in the abovementioned studies were measured at the sites of signal initiation, i.e. close to the cell somata.

The response latency referenced to stimulus onset changes through axonal conduction in the retina and in the optic nerve. Action potentials originating from the peripheral retina propagate through unmyelinated axons along tens of millimeters in humans and many mammalian species until leaving the eye [15]. Ganglion cell-type specific conduction velocity may contribute to spike timing differences. Based on antidromic electrical stimulation early studies in the cat suggest that X- and Y-cell axons conduct at different velocities within the retina [16], [17]. The distinction between X- and Y- cells is prominent in the myelinated optic nerve, where Bishop and co-workers [18] established the separation of the rapidly and slowly conducting axons. Thus, the optic nerve may be another source of temporal dispersion among action potentials.

**Figure 1:**
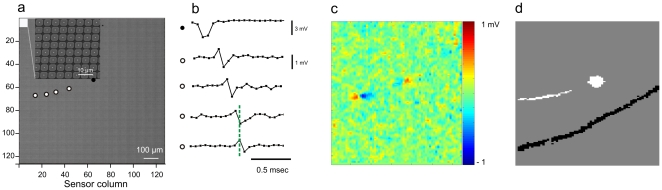
‘Electrical images’ of retinal ganglion cells and axons of passage mark the action potentials' propagation path. (**a**) Low-magnification electron micrograph of the sensor array used to record the electrical activity of retinal ganglion cells and corresponding axons. The sensor array comprises 128×128 extracellular recording sites in 1 mm^2^. The inset shows individual recording sensors at higher magnification. The filled black circle marks a representative sensor where a somatic signal is recorded (shown in b). The four open circles mark representative sensors that record the axonal signals shown in (b). (**b**) Somatic signal with extracellular negative voltage deflection recorded underneath a retinal ganglion cell soma (uppermost trace, marked by filled circle). Each filled symbol of the trace marks the recorded voltage at one sampling point in time. In the 0.5 msec following the somatic signal many biphasic signals are recorded by extracellular sensors: four of them are shown in the lower traces (open circles to the left of traces corresponding to recording sites in (a)). The temporal biphasic signal indicated by the green dashed line corresponds to the spatial biphasic voltage change shown in (**c**). Each image pixel in (**c**) represents the voltage recorded at one time point (corresponding to the green dashed line in (b)) on the corresponding sensor. The color bar indicates the calibrated extracellular voltages (see Methods). (**d**) The electrical footprint of a retinal ganglion cell soma and its proximal axon mark the action potential propagation path. The white image is calculated as the sum of those sensors that record the extracellular action potential (see Methods) shown partially in (b) and (c). In addition the sensors recording biphasic action potentials within an axon of passage are shown in black. These sensors delineate the electrical footprint of an axon of passage. Individual electrical images from this axon are shown in (Fig. 2b).

In this study we investigated how intraretinal conduction in the rabbit retina changes the relative timing among spikes from different ganglion cells. We ‘imaged’ the initiation of light-evoked action potentials and their orthodromic propagation along intraretinal axons using a multi-transistor array (**Fig. 1a**) [19], [20], [21] that provides high spatial and temporal sampling (7.4 µm at 8.2 kHz). We relate the intraretinal conduction time for different ganglion cells to their response latency after flashed stimuli. Finally we discuss the effect of extraretinal conduction variability based on published data [17], [22], [23], [24], [25], [26].

## Materials and Methods

All experimental procedures were carried out in compliance with the institutional guidelines of the Max Planck Society and the local government (Regierung von Oberbayern; Statement of Compliance #A5132-01). All animals are sacrificed prior to the removal of organs in accordance with the European Commission Recommendations for the euthanasia of experimental animals (Part1 and Part 2). Housing and euthanasia of the rabbits are fully compliant with the German and European applicable laws and regulations concerning care and use of laboratory animals.

### Semiconductor chips

The semiconductor chips used to record ganglion cell activity have been described in a recent publication [20]. Briefly, the chips were wire bonded to standard ceramic packages (CPGA, Spectrum, San Jose,CA). A custom-made Perspex chamber with an inner area of 12 mm^2^ was attached to shield the bond contacts and to expose the multi transistor array to culture medium. The chips were gently cleaned with detergent (Tickopur R36, 5%, Stamm/Berlin, 80deg C), and rinsed with ultra-pure water (resistivity: 18 MΩ cm). After drying in a nitrogen stream we applied ∼500 µl of a solution of a 1 mg/ml poly-L-lysine (P1399, MW 150 −300 kDa, Sigma, Germany) dissolved in ultra-pure water. After leaving this solution in place for at least 2 h, the chips were rinsed with oxygenated Ames medium (A 1420, Sigma, Germany) prior to positioning of the piece of retina.

### Multi-transistor-array recording

The electrical response of the retina can be measured with an array of 128×128 equally spaced sensor transistors covering an area of 1 mm^2^ at a sampling frequency of 4.1 kHz. As a default configuration we recorded from every other column of sensors, concomitantly increasing the sampling frequency to 8.2 kHz. Occasionally we recorded an area of 32×128 sensors, which allowed us to increase the sampling frequency to 16.4 kHz. The read-out scheme creates a time gradient along the sensor area, as only 16 sensor transistors are read out at exactly the same time. For the calculation of conduction velocities we corrected for these time shifts.

Transistors were calibrated by applying an AC voltage (frequency: 70 Hz; amplitude: 3 mV peak-to-peak) to the bath electrode [19], [20]. The calibration voltage gives rise to a change of the electrical potential at the surface of the chip. The local change of electrical potential couples through the insulating electrolyte/chip interface to the top metal contact and to the gate of the transistor and proportionally modulates the source-drain current. Conversely, during an experiment, the ion currents through excited retinal ganglion membranes change the local extracellular voltage with respect to the bath electrode. The potential at the chip surface couples through the insulating electrolyte/chip interface to the gate and proportionally modulates the source-drain current. The response of a transistor is solely determined by the potential above the insulating TiZrO_2_ averaged over the diameter of the top contact. In the presented experiments the insulating TiZrO_2_ had a thickness of ∼30 nm. The chip read-out pattern was optimized to avoid cross-talk of transistor signals. During the recording, the columns of the sensor array were sequentially connected to 128 line amplifiers. After a settling time of 720 nanoseconds, the output of these line amplifiers was multiplexed over another 640 ns into 16-output channels. The read-out time of 128×64 sensor array was therefore ∼88 µs. Within each sensor column, an 8∶1 multiplexer selects 16 sensors (sensor spacing 125 µm) that are read out within ∼80 ns.

### Identification of action potentials and assignment to the corresponding ganglion cells

The method for identifying action potentials using the multi-transistor array and assignment to corresponding neurons has been described in two recent reports [20], [21]. Briefly, the identification of retinal ganglion cell action potentials is accomplished in three steps: (a) Identification of threshold crossings of a signal vector *V* calculated from neighbouring extracellular voltages, (b) Assignment of threshold crossings to one action potential and (c) Assignment of action potentials to corresponding neurons. We refer to the above cited publication for details of each individual step.

With respect to the identification of threshold crossings (*step (a)*) we emphasize that for each recorded data point the length of a signal vector *V* is calculated as: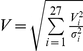, with 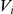 representing the signal amplitude of data point *i* in neighbourhood, 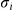: root mean square (*rms*) noise of transistor in neighbourhood. The sum runs over a 3×3×3 neighbourhood (3 sensor rows, 3 sensor columns, 3 time points) surrounding the data point under consideration. The data point itself is part of the neighbourhood. If 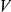 exceeds a threshold of 15 the data point is saved and considered part of the extracellular waveform that represents the action potential. Assuming equal noise on each of the nine neighbouring sensors and homogenous coupling on these sensors the threshold value of 15 means that those extracellular voltages exceeding 15/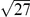
×*rms* of the corresponding sensor are detected. This threshold value is close to that of a previous study using metal electrode arrays (2.5×*rms* in [27] but slightly higher than the threshold used for dissociated rat neurons [20]. The higher threshold was selected to avoid the detection of somatic signals together with axonal signals, as discussed below. In a supplementary figure (**Fig. S1**) we confirm that the somatic signal waveforms recorded from retinal ganglion cells with the multi-transistor-array are similar to those recorded with a multi-electrode array by one of the authors in a previous study [27]. Signal amplitudes differ (**Fig. S1**); however, this does not influence the spike-sorting technique. The spike sorting is done offline and semi - automated. The final spike trains are tested to obey a refractory period of at least 1 millisecond. No action potentials with interspike intervals shorter 1 millisecond were assigned to one neuron using the described method. To confirm proper spike assignment we display the calibrated voltage traces from seven sensors that record nearby ON ganglion cells including the fast cell in supplementary figure **Fig. S2**.

The presented algorithm [20] detects somatic signals only. We find empirically that the signal amplitudes of axonal signals are ∼10 times smaller than those of somatic signals. Axonal signals were identified by inspection of somatic signals and the electrical images following these somatic signals (**Fig. 1 b–c**, [28]). If one somatic signal was followed by a biphasic signal, then all somatic signals of this neuron were followed by axonal signals. Thus, the axonal signals and conduction velocities from proximal axon segments could be identified sampling the sensor positions recording a ganglion cell's maximal amplitude within one piece of retina (**Fig. S2**). Signals from axons of passage were identified using a smaller threshold value (threshold = 11.7, see [20]) compared to the value (threshold = 15) used for somatic signal detection. After the detection, threshold crossings were continuously monitored within floating windows of 2 milliseconds. Visual inspection of this activity allowed detecting electrical footprints of axonal propagation (**Fig. 1 d**). The separation between individual axons is based on differences between electrical footprints.

To calculate the conduction velocity we considered voltage maps of the sensor array that were filtered with a Gaussian spatial filter (width 8 µm). The identification of action potentials was performed using custom routines written in C++ and LabView (National Instruments). The calculation of the propagation velocity and spike train cross-correlation was performed using routines written in MATLAB.

### Preparation of the retina

Experiments were performed on whole mount rabbit retinas in accordance with the animal use committee of the Max Planck Institutes. The preparation of the retina follows previous reports [29]. Briefly, rabbits (strain New Zealand White, 8–12 weeks of age, Charles River) were dark adapted for one hour, anesthetized by intramuscular injection of ketamine (50 mg/kg) and xylazine (5–10 mg/kg) and euthanized by a mixture of embutramide, mebenzonium iodide and tetracaine hydrochloride (T-61, Intervet, Unterschleißheim,Germany). The enucleated eye was hemisected under dim red light and the retina peeled off the sclera. Pieces of retinas (∼4×4 mm^2^) inferior to the visual streak were cut under a dissecting microscope, measuring at the same time the location relative to the myelinated band using a microscopic ruler etched on a cover glass (Olympus). The retinal portions were transferred to the chip chamber and mounted ganglion cell side down, on the coated multi-transistor array. The retina was held in place during the experiment using a custom made ring covered with a dialysis membrane.

### Visual stimuli

Visual stimuli were generated using Visionworks software (Vision Research Graphics, Durham, NH) and presented on a miniature monochrome organic light emitting diode display (OLED; eMagin Corp., Bellevue, WA; mean irradiance at the retina 9 mW/m^2^, resolution, 800×600 pixels; 60 Hz refresh rate) illuminating the back focal plane of a 5× objective (LMPlan Fl; Olympus Optical, Tokyo, Japan). The monitor is mounted on an upright microscope BXW50 (Olympus Optical, Tokyo, Japan) in place of the video/photo output using a custom-made adapter.

We flashed spots of different sizes centered onto the retina. We presented stimuli using two protocols, with spots of either higher or lower intensity than background. The spots were presented for 300 milliseconds interleaved with a homogenous background presented for 200 milliseconds. The first protocol excited ON and ON-OFF cells, while the second excited OFF and ON-OFF cells. The background light intensity (*I*_BG_) was set at 9 mW/m^2^ (photopic/mesopic range), measured at the location of the retina (Optical Meter 1835-C, Newport Spectra-Physics, Darmstadt, Germany). The stimulus contrast was calculated as the ratio 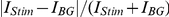 where *I*_STIM_ represents the stimulus intensity. The contrast is the same for ON and OFF stimuli.

A second stimulus consisted of a 16×16 pseudo-random flickering checkerboard, as previously used for receptive field mapping [29]. The refresh rate of the checkerboard stimulus was 30 Hz and the size of each square was 75 µm (total size of the stimulating field was therefore 1.2 mm^2^). In this study the stimulus was used to test the relative response latency of two simultaneously stimulated ganglion cells. Estimation of the ganglion cells' receptive fields using spatial flicker stimuli is prohibited currently by the large amount of data (∼150 Megabyte/sec) recorded with the multi-transistor-array.

### Identification of physiological ganglion cell types

Direction selectivity was tested using a square wave spatial grating (spatial frequency 1 cycle/mm, velocity 2 mm/sec) moved in eight equally separated directions. The spatial extent of the moving grating was ∼1200 µm on the retina thus stimulating all ganglion cells at once. For each direction the population response was recorded for 10 seconds and an average firing rate calculated. Direction selective cells showed a characteristic tuning curve in their firing frequencies [29], [30].

Transient and sustained cells displayed similar firing frequencies for any direction of the different moving gratings. Transient and sustained cells were identified based on their response properties to flashed stimuli (500 µm spot diameter). The firing rate of transient cells (of both ON and OFF type) to flashed spots decayed below 20% of their maximal value within 100 msec. Sustained cells (of both ON and OFF type) maintained a constant firing rate above the 20% level throughout the stimulus presentation time (300 ms).

Intra-burst intervals are calculated in the order they occur in a train of action potentials. As described in a previous publication [29] we calculate the median first intra-burst interval if there is a period of silence of 25 milliseconds preceding one spike and the second spike occurs within the next 25 milliseconds. The second intra-burst interval is calculated as the median interval between the second and third spike in a burst.

### Temperature control

The temperature of the oxygenated Ames' medium (32–34°C) medium was monitored using a thermometer (Pt 100) immersed in the recording chamber. At the same time a peltier element cooled the chip, which would otherwise heat the retina above the physiological range.

## Results

To follow the propagation of visually evoked action potentials in retinal ganglion cell axons, we interfaced portions of the rabbit retina with high density multi-transistor arrays [19, Lambacher, 2011 #367] in a whole-mount configuration. The retina was placed ganglion cell side down to enable simultaneous recording of extracellular voltage changes with up to 16384 extracellular sensor sites packed into 1 mm^2^ (**Fig. 1a**).

### Electrical images of action potentials propagating through retinal axons

In this study we recorded the extracellular voltages of retinal ganglion cells stimulated with different light stimuli. The signals were calibrated in terms of the average extracellular voltage at the oxide surface of the recording sensor (see *Methods*). Somatic signals (upper trace, **Fig. 1b**) are identified by a negative voltage deflection attributed to sodium influx during the action potential rise phase [31]. Somatic signals on one sensor were often followed in time by signals with a biphasic shape (**Fig. 1b**) on neighboring sensors, which we attributed to axonal signals [31]: the leading positive voltage deflection is attributed to the capacitive load of the axonal membrane along the signal propagation direction followed by the negative voltage deflection due to sodium influx. Somatic (**Fig. S1**) and biphasic signals resemble recordings using metal multi-electrode arrays [28], [32]. The temporal biphasic signal corresponds to a spatially biphasic signal recorded on many adjacent sensors. This spatial activity map (**Fig. 1c**) represents the “electrical image” [28], [33], [34] of one action potential at one given time point.

**Figure 2:**
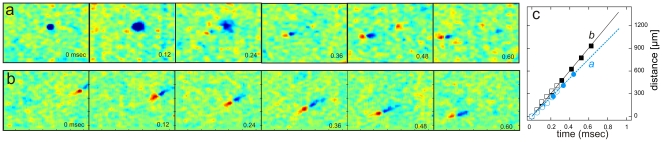
Consecutive electrical images allow for precise calculation of axonal conduction velocity. (**a**) Recording of a somatic action potential (blue area in the first voltage map) followed by biphasic signals propagating along the proximal axon. The electrical image at time point 0.36 msec corresponds to Figure 1c. Note that only part of the1 mm^2^ array is shown. (**b**) Recording of biphasic voltage signals from an axon of passage. The footprint of this axon is shown in Fig. 1d (**c**) The distance between consecutive capacitive peaks of the action potential (red color) scales linearly with the time elapsed between consecutive time frames. Filled symbols represent data from measurements at 8.2 kHz (shown in **a** and **b**) while the data represented as open symbols were calculated from recordings at 16.4 kHz.

To reconstruct the footprint (**Fig. 1d**) of a propagating action potential on the array we summed consecutive electrical images in a time interval of 1 msec following a somatic spike. Sensors recording voltage deflections above threshold (*see Methods*) due to occasional correlated activity from other neurons are eliminated by averaging one hundred ‘electrical images’ and by thresholding the result at 30%. This threshold is higher than the maximum percentage of correlated activity encountered among ganglion cells [35], [36] and thus separates the interneuronal from the intraneuronal sensor correlations. The selected sensors represent the path of the action potential across the recording array (**Fig. 1d**). They reflect the signal propagation along the proximal axon. In addition to action potentials elicited by ganglion cells in the recording area we measured biphasic signals propagating across the array. Electrical footprints for these axons of passage are found in a similar way as described above. We start with a sensor recording one temporal biphasic signal (**Fig. 1b**). Within the following 1 msec before and after this axonal spike all active sensors (i.e. sensors that detect threshold crossings) are summed. Occasionally sensors from other axons or cell bodies are recorded, due to the smaller threshold value (see *Methods*). To eliminate sensors that record activity from other neurons hundred axonal footprints are averaged and those sensors are eliminated that were active in less then 30% of the recordings (**Fig. 1d**, black elongated area). The measurement of conduction velocity along the two axon footprints is shown in **Fig. 2**.

Using time-consecutive electrical images we evaluated the propagation direction and conduction velocity along 127 intraretinal axons in 7 retinas. The spatial locations of the axonal capacitive peaks marked by the positive voltage deflections in consecutive time windows were used to measure the conduction velocities in proximal axons (**Fig. 2a**) and in axons of passage (**Fig. 2b**). Plotting the travelled distance by one action potential versus elapsed time results in a linear relation where the slope represents the axon's conduction velocity (**Fig. 2c**).

To compare conduction velocities among multiple cells we first estimated the precision of our measurement procedure. We estimated the conduction velocity for subsequent action potentials elicited by the same cell. The standard error for the mean conduction velocity of each cell or axons of passage was less than 20 mm/sec. All actions potentials were faithfully conducted up to instantaneous firing frequencies of 500 Hz (inset in **Fig. 4a**).

The electrical images presented in Fig. 2 allow identifying the orthodromic propagation of action potentials towards the optic nerve head. However, since the recordings were performed in an ex vivo preparation, antidromic signals may occur and potentially contaminate the results. We measured in two preparations with rather small retinal pieces antidromic spikes immediately after interfacing the tissue onto the recording array (**Fig. S3**). We interpret this finding as a preparation artefact – possibly due to perturbed intracellular ionic concentrations. Antidromic spike were not observed in any of the recordings presented below. As we never measured antidromic spikes following orthodromic propagation we infer that refractoriness leads to spike dissipation at the sealed axon ending. There are no receptors available to depolarize the axonal ending and elicit backpropagating action potentials.

### Conduction velocities at different eccentricities

**Figure 3:**
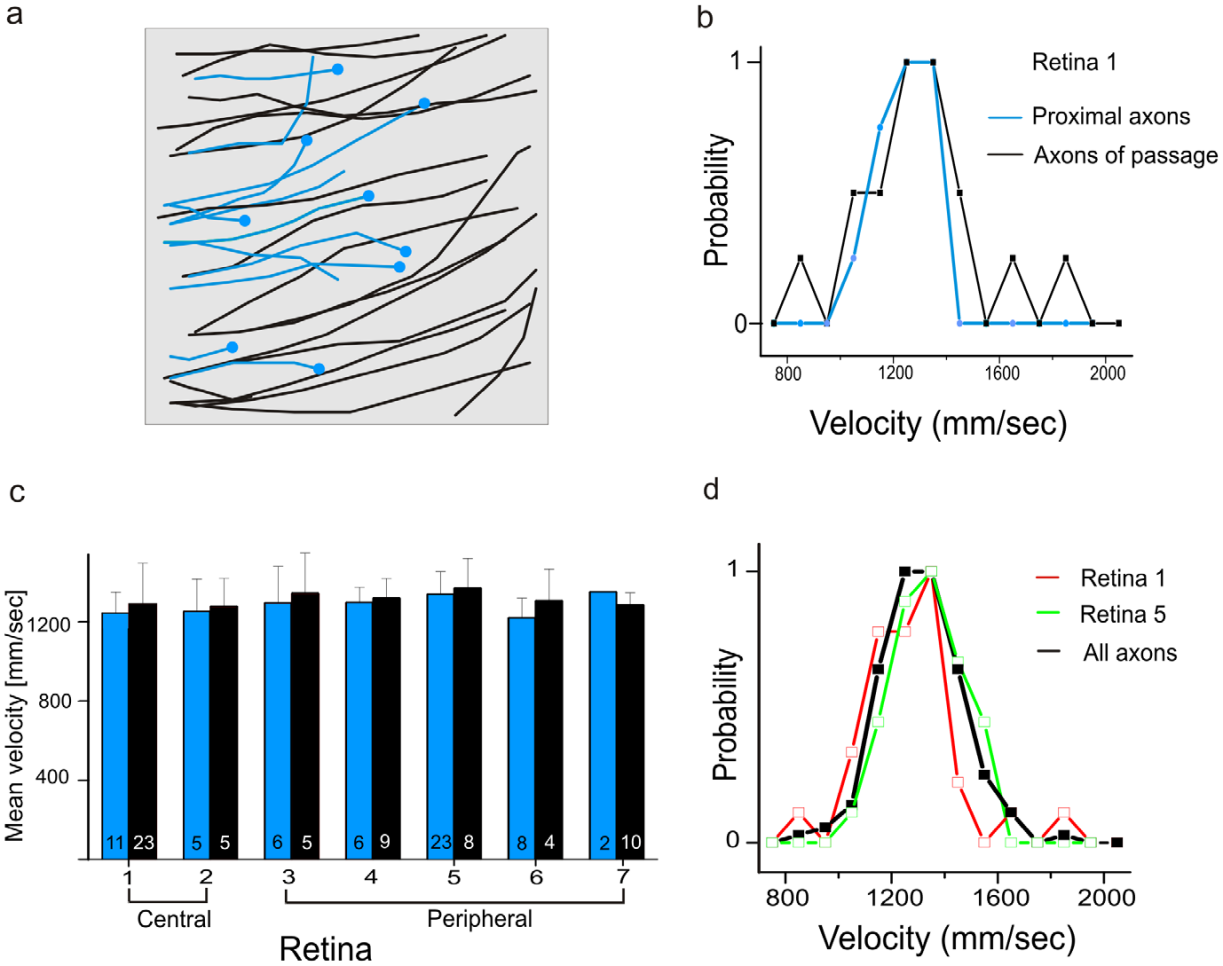
Mean velocities measured in small retinal portions are constant irrespective of retinal eccentricity. (**a**) Schematic pathways of axons of passage (black) and ganglion cell somata with proximal axons (blue) in a 1 mm^2^ portion of the retina. (**b**) The velocity distributions for the proximal axons (blue) and for the axons of passage (black) shown in (**a**) are similar. Mean conduction velocities are 1.24 m/sec for proximal axons (n = 11) and 1.28 m/sec for axons of passage (n = 23). (**c**) Mean conduction velocities calculated for seven portions from different retinal eccentricities. The first two portions were recorded close to the visual streak, the following portions from the retinal periphery. Blue bars indicate mean velocities calculated for proximal axons; black bars correspond to mean velocities of axons of passage. The numbers on each bar indicate how many axons were considered for calculation of the mean. Error bars indicate the standard deviation of the mean value. (**d**) The distribution of all measured velocities (black curve) and the velocity distributions in two selected retinal portion (red curve summarizes the velocities from (b), green curve the velocities from retina 5) are unimodal with similar mean values. The red curve summarizes the velocities from a central portion (shown in b), the green curve the velocities from a peripheral retinal portion.

To determine if the conduction velocity varies with retinal eccentricity, we first compared the velocities of all axons originating within one piece of retina with velocities of axons passing through that area. The size of the measured portion is determined by the sensor array size of 1 mm^2^. In the presented example (**Fig. 3a**) we identified 34 axons, 23 of which were axons of passage and 11 of which were proximal axon segments (**Fig. 3a**). The mean velocities for these two populations are: 1240±100 mm/sec, mean ± std (n = 11 proximal axons) and 1290±200 mm/sec (n = 23 axons of passage). The two populations are not significantly different (0.05 level, two-sample t-test, p-value = 0.3) suggesting that conduction velocity does not vary with eccentricity (**Fig. 3b**). Because the interfaced retinal portions (4×4 mm^2^) on the sensor array (1 mm^2^ array) are rather small the cell somata from the axons of passage may not lie far outside the measured area. To test more rigorously the finding that conduction velocity is constant across the retina we selected samples from different retinal eccentricities using a microscopic ruler during the preparation (*Methods*). We compared the mean velocity of axons originating in the periphery (>10 mm inferior to the visual streak) to axons originating closer to the myelinated band (<5 mm inferior to the visual streak). We found that the mean values across different retinas and eccentricities are not significantly different at the 0.05 level (two-sample t-test, **Fig. 3c**). Note, that we were not able to record axons close (0–2 mm) to the optic nerve head where the rabbit retina thickens and intraretinal axons are myelinated [37].

Although the mean velocity does not scale systematically with eccentricity, we do measure in each retinal patch a distribution of velocities (**Fig. 3b** and **Fig. 3d**). The pooled velocity distributions of proximal axons and axons of passage in individual pieces of retina are unimodal (central retina 1: 1280±184 mm/sec; mean ± SD, n = 34 axons; peripheral retina 5: 1330±146 mm/sec; n = 31 axons). Finally we calculate the average velocities for all 127 axons in all retinas (1300±150 mm/sec; n = 127).

These results demonstrate that velocity tuning is eccentricity-independent. The unimodal distribution found in each portion indicates that physiological cell types are not separable in the rabbit retina based on their intraretinal conduction velocity alone.

### Conduction velocity for different ganglion cell types

We used classic visual stimuli (flashed spots and drifting gratings) to identify physiological ganglion cell types (*Methods*). For each identified cell type we specify the average intraretinal conduction velocity calculated within different retinas.

**Figure 4:**
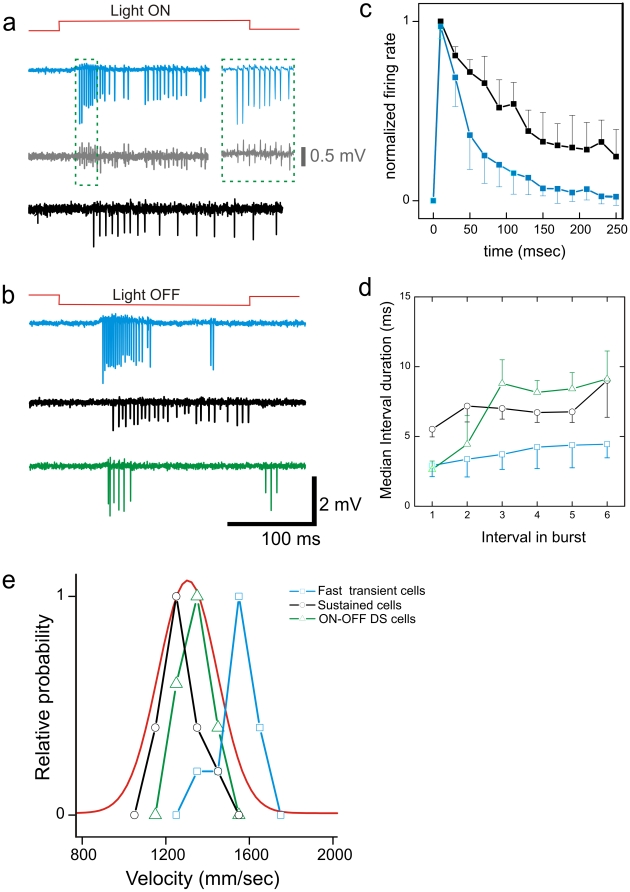
Average velocities of different ganglion cell types. (**a**) Selected ganglion cell responses to flashed spot stimuli from the same retinal portion. From top to bottom: Fast responding ON cell (blue), axonal recording of the same cell on a different sensor (gray), sustained ON cell (black). The inset demonstrates that every somatic spike is reliably transmitted along the axon. (**b**) Selected ganglion cell responses to flashed OFF stimuli from the same retinal portion shown in (a). From top to bottom: Spike trains of fast responding OFF cell (blue), sustained OFF cell (black) and ON-OFF cell (green). (**c**) Poststimulus time histograms for the fast responding cells and the sustained cells. Time bin 20 msec. (**d**) Spike patterns calculated for the fast responding transient cells (ON and OFF cells pooled), the sustained cells (ON and OFF pooled) and the ON-OFF direction selective cells. The spike pattern is calculated as the ordered intra-burst interval for each spike train of a specific cell type (see description in Methods section). (**e**) The velocity tuning of each of the three cell types is narrower than the tuning of the whole population (red). Each tuning curve was normalized to its maximum value. The velocity distribution for all axons (red) was approximated by a Gaussian distribution.

**Figure 5 pone-0020810-g005:**
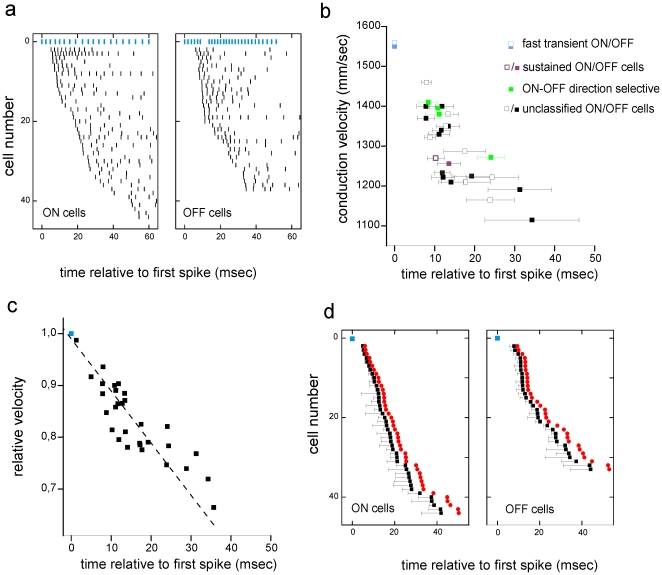
Velocity tuning changes relative response timing along intraretinal conduction. (a) (Left panel) The response of 44 ON cells to a flashed bright spot. Each tick marks the occurrence of one spike. Time was measured with respect to the occurrence of the first spike after stimulus onset. The blue ticks mark the spikes of the fast transient ON cell (see also Fig. 4a). (Right panel) The response of 34 OFF cells to a dark spot. The OFF cells were recorded in the same retinal portion as the ON cells. Each tick marks one action potential. Blue ticks mark the spikes of the fast transient OFF cell. (**b**) The fast responding transient cells conduct with higher velocity than the slow responding cells. A linear relation between conduction velocity and response delay with respect to the first spike is obtained for 17 OFF cells and 11 ON cells of the population shown in (a). Three ON-OFF direction selective cells and two sustained cells recorded in this retina conduct with similar velocity. (**c**) All conduction velocities were normalized to the velocity of the fast conducting cell. The linear relation between relative conduction velocity and response delay of the first spike is used to estimate the dispersion for populations of ON and OFF cells. (**d**) The mean delay of each cell's first spike calculated with respect to the fast cell's first spike. The temporal jitter calculated from six stimulus repetitions for each cell is 5 msec on average (black error bar). The difference in conduction velocity increases the average latency (red dots assuming an equal intraretinal propagation distance of 20 mm for all axons in the portion under consideration.

Fast responding cells precede the population response of all ganglion cells in response to flashed spots (1 mm diameter) by a few milliseconds (**Fig. 4a, 4b** and **Fig. 5a**). If the stimulus is repeated it is always the same cell that spikes first. These cells precede the population response if smaller spot sizes (0.5 mm diameter) are used. Fast responding cells with similar electrophysiological properties were found among ON and OFF cells and will not be discussed separately. The firing rate of these cells to flashed spots decayed below 20% of their maximal value within 100 msec (**Fig. 4c**). To further characterize this cell type we evaluated the burst patterns that occurred during spontaneous activity in dim light (see *Methods*). Fast responding transient cells usually fired consecutive spikes with short intra-burst intervals (2–3) msec (**Fig. 4d**). The electrophysiological evidence (**Fig. 4c, d**) suggests that these cells represent so-called ON and OFF brisk transient ganglion cells [22], [29], [38] that are homologues of the Y cells in cat or primate. Fast responding cells that displayed the abovementioned electrophysiological characteristics were recorded in every piece of retina, and in 5 retinas we also measured the concomitant velocities. They conduct with higher intraretinal velocity (1500±50 mm/sec; mean ± std, n = 6, vs. 1300±150 mm/sec average for all cells) (**Fig. 4e**). In one retina we recorded two cells with the same response characteristic and similar conduction velocity in the same retinal portion.

Sustained cells (of both ON and OFF type) maintained a constant firing rate above the 20% level throughout the stimulus presentation time (**Fig. 4c**). The spike trains of sustained cells displayed average intra-burst intervals between 5–10 msec (**Fig. 4c** and [29]. Cells that did not display these two electrophysiological characteristics are not included in the ‘sustained’ class. Sustained cells were recorded in every retinal portion. They conduct with lower intraretinal velocity (1200±50 mm/sec; mean ± std, n = 10) than the fast cells (**Fig. 4e**). For other cell types, such as local-edge detectors that display longer inter-spike-intervals [29], we recorded few proximal axons; these cells thus are not considered here.

ON-OFF direction selective ganglion cells (DSGC), respond best to stimulus movement in the preferred direction and are silent to stimuli moved in the opposite, null direction [30]. ON-OFF DSGCs include four subtypes, each of them sensitive to motion in either anterior, posterior, inferior or superior direction [30]. Cells coding for opposing directions were recorded in the same retinal portion. Their conduction velocities were similar. The conduction velocities of ON-OFF DSGCs did not vary across retinas and did not vary with retinal eccentricity. The conduction velocity distribution (1337±78 mm/sec; mean ± std, n = 11) was narrower for these cells than for to the cell population as a whole (**Fig. 4e**), but did not form a distinct cluster that separated them from other cells, such as the sustained cells (**Fig. 4e**).

Axons of different ganglion cells type conduct with different intraretinal velocities. In the following we compare the intraretinal conduction time to the cell-type specific response latencies measured at the site of action potential initiation.

### Response latency and corresponding intraretinal conduction time

Differing presynaptic circuitry elements may retard spike initiation to a varying degree in the different ganglion cell types, in response to the same visual stimulus. To test how differences in response latency are changed by differences in propagation velocities we simultaneously examined the response delay and the conduction delay to flashed spot stimuli.

Under our experimental conditions (see *Methods*) the first spike in the ganglion cell population appeared ∼50 msec after stimulus onset. In this study we disregard this latency common to all cells and consider relative latencies only. Relative latencies were calculated for each cell's first spike with respect to the first spike of the fastest responding cell (**Fig. 4a** **,** **Fig. 5a**). We found that relative latencies ranged from 5 to 60 msec (20±10 msec, mean±std) within a population of 44 identified ON cells stimulated with spots of 1000 µm diameter and 0.3 contrast (see *Methods* for contrast definition). The same range of relative response latencies was measured for 34 OFF cells (**Fig. 5a**). The response variation increased for stimuli with lower (0.15) contrast (23±14 msec, mean ± std) and slightly decreased to for stimuli with higher contrasts (0.81) respectively (16±9 msec, mean ± std). A fraction of cells (∼50%) of the same ganglion cell population was stimulated by smaller spots (500 µm diameter). Within that fraction the same fast responding cell always led the population response. The order of responding cells in the population response changed slightly for smaller spots. Because the number of responding cells differed for the two protocols (500 µm vs. 1000 µm stimulus) the change in rank order will not be further investigated. Stimulation with larger flashes (1500 µm diameter) elicited similar response patterns to those of the standard protocol with 1000 µm (n = 2 retinas). In summary, independent of the flashed stimulus protocol used, it was always one single retinal ganglion cell that spiked first. This provided a reference against which population latencies were measured.

Next, we related the ganglion cell response latencies (1000 µm stimulus diameter, contrast: 0.3) to the intraretinal conduction velocities. The velocities and relative response latencies from a population of 11 ON and 17 OFF ganglion cells recorded in the same retina are shown in **Fig. 5b**. The cells represent a subpopulation of the ganglion cells shown in **Fig. 5a**. Note that only a subset of cells within this preparation could be assigned to one of the RGC subtypes shown in **Fig. 4e**. The response latency was measured for 2 fast transient cells, 4 ON-OFF DS cells and 2 sustained cells (**Fig. 5b**). All ganglion cells that respond after the fast transient cell conduct with a lower velocity. For both populations (ON and OFF cells) we find a negative correlation between conduction velocity and relative response latency: the later the ganglion cell response the slower its axonal conduction velocity. We highlight four ON-OFF DS cells measured in the same retina. They responded with ∼10 msec after the fast cells with little jitter (2–3 msec). The conduction velocities of the axons of passage are not considered here because we could not elicit clear stimulus responses in the corresponding cell bodies outside the array. A second characteristic of the relation between response latency and conduction velocity is the jitter variability. Temporal jitter is calculated as the standard deviation of the relative response latency for repetitive stimulus presentations. Those cells that conduct with relatively high velocity display the smallest jitter, indicated by the small standard deviation of the response latency (**Fig. 5b**). To estimate the delay introduced by intraretinal conduction for all cells stimulated simultaneously we approximate the behavior of response latency and conduction velocity by linear regression. We first normalize each conduction velocity dividing it by the conduction velocity of the fastest conducting cell within that retina. The fast transient cells are marked by the blue symbol in **Fig. 5c**. We found a linear scaling (regression coefficient R^2^ = 0.9, p<0.0001) between conduction velocity and response latency (**Fig. 5c**) among a population of 34 ganglion cells. We used this scaling to estimate the conduction delay for all cells in the two populations shown in **Fig. 5a**.

We infer from similar measured conduction velocities in two nearby ganglion cell axons that they have similar intraretinal conduction time. The underlying assumption of equal axon length is reasonable in the rabbit retina, where axons run largely parallel to each other and orthogonal to the myelinated band. In the retinal portions investigated, we measured a small variation in axonal direction (standard deviation ∼10° from mean direction, n = 7 retinas) thus suggesting an equal conduction length for all axons originating from any given area. Assuming for all cells from one retinal portion an identical intraretinal conduction distance of 20 mm we calculated the intraretinal conduction time. Different conduction times lead to an increase in relative delay between first-spike times in the range of 1.5–10 msec (**Fig. 5d**). The error bars in Fig. 5d mark the temporal jitter of a cell's first spike to stimulus repetitions. The small jitter indicates that during the experiment the response latency is robust. In summary, different intraretinal conduction velocities retard the spike times of all cells referenced to the first spike of the fast cell.

**Figure 6:**
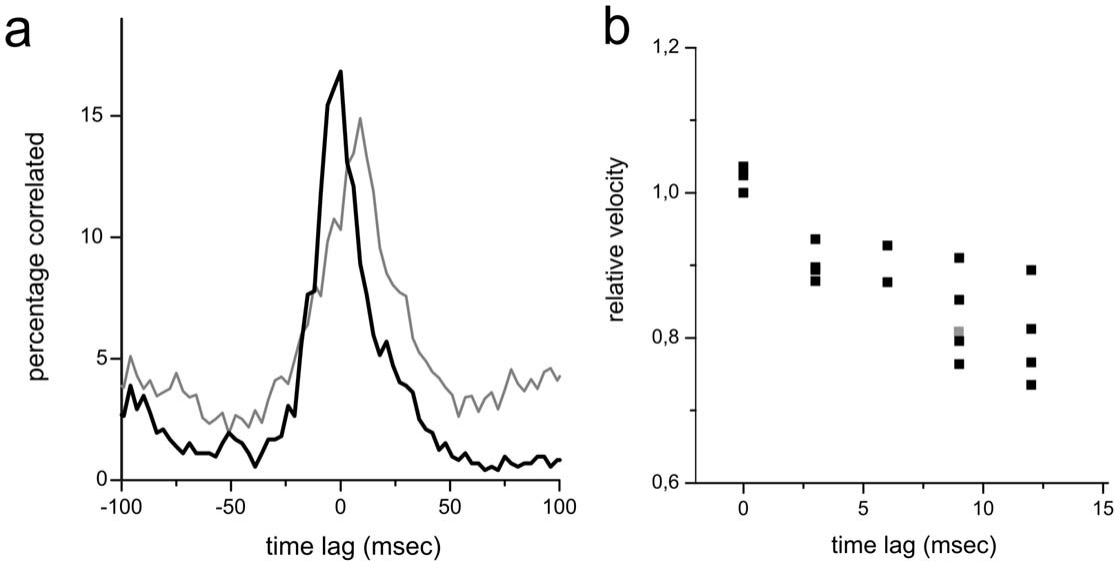
White-noise stimulated ganglion cells reveal that fast responding cells conduct faster than slow responding ganglion cells. (**a**) The cross correlation function for two pairs of retinal ganglion cell spike trains. For one cell pair (black) there is no time lag between the maximal response probabilities of the two cells. For the second pair (gray) cell 1 spikes on average 9 ms earlier than cell 2. All ganglion cells are simultaneously stimulated by the same white noise stimulus (see Methods). Bin Size: 3 ms. (**b**) The relative velocity is calculated for each cell pair. The corresponding time lag (see A) for the same cell pair is larger the smaller the relative intraretinal velocity. Gray symbol denotes the cell pair shown in (a).

In an additional experiment we visually stimulated and recorded the population of ganglion cells presented in **Fig. 5** using a pseudo-random checkerboard stimulus (‘white-noise’ stimulus, Methods). The recorded spikes of individual ganglion cell spikes were pair-wise correlated (**Fig. 6a**). A positive peak time lag indicates that cell 1 (slow conducting cell) spikes 9 milliseconds after cell 2 (fast conducting cell). The time lags were evaluated for those cell pairs where the intraretinal conduction velocity was measured too. We found a linear scaling (regression coefficient R^2^ = 0.7, p<0.0001) between conduction velocity and time lag (**Fig. 6b**) among a population of sixteen ganglion cell pairs. This experiment further supports our conclusion that visually evoked spikes elicited by fast responding ganglion cells leave the eyeball earlier than spikes from the late responding cells.

## Discussion

In this study we measured the propagation of light-induced action potentials in single unmyelinated axons of rabbit retinal ganglion cells using a high-density multi-transistor array. We measured in each piece of retina a distribution of velocities among ganglion cells of different types, with all ganglion cells of a given type conducting at similar velocity. The intraretinal conduction velocities display a negative correlation with the ganglion cell response latencies to flashed stimuli. Thus, the relative timing in population signals originating from nearby cells of the same type will remain largely unaffected by intraretinal axonal transmission, while the relative timing between signals from different nearby cells will be slightly dispersed. In the following we compare our results with previous studies on intra- and extraretinal conductions and discuss the functional implications of the abovementioned correlation between response latency and conduction velocity.

### Measurement of conduction velocity in single retinal axons

From a technical perspective the recording of extracellular axonal signals using capacitive coupling to field-effect-transistor is possible because of the high extracellular resistivity of the tissue [39] and its close contact with the recording sensor (Zeitler, Zeck and Fromherz, *unpublished*). The direction of axonal propagation but not the velocity has been recently estimated in the primate retina by multielectrode arrays with a far larger inter-electrode distance than our system [28]. The high spatial density and high-resistance tissue allows for precise measurement of the conduction velocity at various retinal eccentricities. Early studies in cat and rabbit retinas were mostly restricted to central retinal areas [22, Stanford, 1987 #209]. Multi-transistor arrays thus represent a useful tool for investigating action potential transmission in individual axons in appropriate preparations.

### Unimodal axonal conduction velocity distribution within each retinal portion

Classical cable theory suggests that conduction velocity scales with the square-root of the unmyelinated axon diameter [31], [40]. The conduction velocity distribution described here is consistent with an electron microscopic study in the rabbit retina that reports a unimodal axon diameter distribution irrespective of retinal eccentricity [37]. Similar diameter distributions have been reported for retinas or optic nerve sections of other species [41], [42], [43] with the exception of the bimodal distribution in the cat optic nerve [44].

In the rabbit retina a slight increase of the mean axon diameter towards the periphery was reported. We could not confirm a corresponding increase – however we were not able to record axonal propagation in the myelinated intraretinal band containing the thinnest axons [37]. A discrepancy between mean axon diameter increase and constant velocity has been reported in early studies in the cat retina [17], [45].

Many electron-microscopic studies including the rabbit [37] report a skewed distribution with numerous thin axons and few thick ones. We did not measure such a skewed distribution of conduction velocities in any retinal portion (**Fig. 3d**). This indicates that either conduction velocity does not scale with the square-root of the unmyelinated axon diameter [40], [46] or that our sensor array is more sensitive to large axons. Out of the 100–1000 axons/mm^2^ [37] we recorded only a subpopulation (10–34 per retinal portion, **Fig. 3c**). Our data may not reflect the real intraretinal velocity distribution but may be biased for large (and fast) axons.

### Maintenance of response latencies in a population comprising one cell type

For a ganglion cell population comprising neurons of the same type (fast responding cells, sustained cells, or ON-OFF DS cells), we have shown that their conduction velocity does not vary with retinal eccentricity. The results are in line with earlier evidence in the cat and monkey retina [17], [24] that intraretinal conduction velocity is constant for a given cell type. We cannot confirm results suggesting that velocity increases with eccentricity [16] or is variable within one cell class [25].

Our results suggest several principles governing the relative timing of ganglion cells that, first, intraretinal conduction does not change the relative spike timing difference between nearby cells of the same type. Second, ganglion cells of the same type separated by large distances on the retina may elicit synchronous spikes but exhibit different intraretinal conduction time. It has been shown in the cat that intraretinal conduction difference of spatially separated X-type ganglion cells is minimized by extraretinal conduction [25]. In the rabbit the extraretinal distance between optic nerve head and postsynaptic target (i.e. superior colliculus) is ∼38 mm [26]. Assuming that two extraretinal axons conduct with 10 and 30 mm/ms respectively [26] we obtain an extraretinal conduction time difference of 2.6 ms. This value translates to a ganglion cell separation of 3.4 mm (20° visual angle) assuming an equal intraretinal velocity of 1.3 m/sec. The timing difference for ganglion cells of the same type separated by less than 3.4 mm could therefore be compensated by extraretinal conduction in the rabbit. This result is in agreement with the study by Stanford in the cat retina [25].

What is the functional relevance of the constant time differences among ganglion cells of the same type? It has been shown recently that the relative timing among ON-OFF cell pairs [13] stimulated with spatially inhomogeneous images provides information about the stimulus structure. Our results indicate that these differences are not changed but persist along the axonal conduction. Retinal ganglion cells of the same type are known to exhibit synchronous activity in the dark and also in response to different visual stimuli [47], [48], [49] (**Fig. 6**) if they are separated by less than 1 millimetre [47], [49]. Synchronous activity shared by two neurons conveys 10–20% more information about visual scenes that is inaccessible when individual neural responses are considered [8], [50]. Our intraretinal velocity measurements and comparison with extraretinal studies indicate that synchronicity between cells of the same type is not changed by axonal conduction.

### Dispersion of response latencies in a population comprising different cell types

Populations comprising retinal ganglion cells of different type respond with different latencies to the appearance of a new stimulus (**Fig. 4** **–** [Fig pone-0020810-g005] **6**) and [51].

These latency differences are further enhanced in the rabbit retina through intraretinal conduction. Fast responding cells conduct with higher velocity compared to slow responding cells (**Fig. 5** **–** **6**). Previous studies in the cat retina report that Y cells conduct about two-fold faster (∼5 m/s) compared to X-cells [16], [17, Cleland, 1971 #88]. Such strong conduction velocities differences have not been found in the rabbit retina (this study) or in the monkey retina [24]. However, the correlation between response latency and axonal conduction may be a general feature among different retinas, as Y –like cells respond faster to flashed stimuli than do other cell types [51].

Could the latency differences introduced by presynaptic circuitry and intraretinal dispersion be compensated by extraretinal variability? The time difference between the first spike of the fast cell and the first spikes of other cells was at least 7 msec (**Fig. 5d**) and increased slightly by intraretinal conduction. Such time difference cannot be compensated for by extraretinal conduction (see the estimate of 2.6 ms in previous paragraph).

A second source of variability may be the temporal jitter measured to stimulus repetitions. Single-cell jitter ranges between 1–10 milliseconds depending on stimulus luminance, stimulus contrast and structure [52]. This jitter is smaller for brisk, fast conducting cells than for cells that respond late and conduct slow ((**Fig. 5**) and [53]). When referenced to the first spike of the fast cell rather than the stimulus onset, the relative jitter was small (∼1 msec) for those cells whose response immediately followed the fast responding cells, and up to 15 msec for the slow responding cells (standard deviations in **Fig. 5d**). Thus, it appears unlikely that temporal jitter changes the spike time rank order in the ganglion cell population.

Could the under-sampling of the ganglion cell population influence our conclusion? Even with the high-density recording array we did not identify all ganglion cells in the investigated mid-periphery retinal portion (∼300 cells/mm^2^ [54]). However, the linear relation between response latency and intraretinal conduction velocity (**Fig. 5** **,** **Fig. 6**) is based on numerous data points and unlikely to be changed by additional, missing ganglion cell (types).

Thus, the relative timing in the population response measured at the site of action potential initiation is slightly dispersed by differences in intraretinal conduction velocity among cell types. This dispersion may be neutralized to some extent by differences in extraretinal conduction velocity. However, this is not expected to be sufficient to produce synchronization of responses between the fast cells and the other cell types.

How could the brain use the relative timing in a population response? A series of theoretical studies has proposed that the arrival times of first spikes forms a rapid neural code for the visual system [7], [13], [55] and other sensory modalities as well [5], [10], [11], [12]. Our results suggest that for most cell pairs of different types the rank order of impulses will not be changed by differences in axonal conduction velocity between these cells. Studies on spike latency coding are motivated by the findings that neurons in higher brain areas respond within 100 ms after the onset of a new visual stimulus [56], with a considerable portion (∼50 ms) of this time “lost” in the retina. A caveat in latency coding schemes is the knowledge of stimulus onset. It has been suggested recently that in the auditory system a coincidence detector neuron may integrate the population response [5] and signal a stimulus change, after the population activity crosses a certain threshold. Given the relatively broad distribution of first-spike times within a simultaneously activated retinal population (**Fig. 5a, d**) we propose an alternative. The fast cells may provide the reference point (*time zero*) for the appearance of a new visual stimulus, such as experienced after eye movements [**57**]. Several studies in the cat [23, Weng, 2005 #323] and monkey [58], [59] demonstrate that signals from Y-like cells reach their postsynaptic targets faster than signals from X-like cells. In the rabbit, we have shown that the fast responding and fast conducting cells belong to the brisk transient ganglion cell class. This class is homologous to the Y-like cells [22] and thus a good candidates to act as a ‘visual switch’ [3] for higher visual areas, signalling the onset of a new stimulus.

In conclusion, our study indicates that on one hand differences in spike timing between pairs of rabbit ganglion cells of the same type are not changed by axonal conduction. On the other hand, the broad population response of simultaneously stimulated ganglion cells is slightly dispersed by intraretinal conduction. Most importantly, the first spikes of the fast responding ON or OFF cells reach their postsynaptic targets before the activity of the remaining population of simultaneously activated ganglion cells.

## Supporting Information

Figure S1 **The extracellular voltages near ganglion cell somata are similar for multi-transistor-array and metallic multi-electrode-arrays recordings.** (a) Extracellular voltage traces recorded with a multi-transistor-array (sampling frequency 16 kHz). Thick black trace represents the average waveform. (b) Extracellular voltage traces recorded with a multi-electrode-array (Zeck and Masland, 2005). Thick black trace represents the average waveform (sampling frequency 30 kHz). (c) The two average waveforms from (a) and (b) were normalized to the individual maximal value. The shapes of the waveforms are similar for the two recording systems.
(TIF)

Figure S2 **Calibrated voltage traces of seven nearby ON cells reveal relative timing differences.** (a) (Left) The positions of 44 sensors that recorded ON ganglion cells in one retinal portion (spike times are shown in **Fig. 5a**). These positions were assigned to the corresponding ganglion cells by the custom-written spike-sorting program. (Right) Zoom of one part of the array with nearby ON cells. (b) Calibrated extracellular voltage traces showing seven nearby ON cells marked in (a). Trace 1 is from the fast ON cell (**Fig. 4a**). All voltage deflections are clearly separated. Trace 2 shows the recording from an ON cell that is ∼36 µm away from the sensor recording the fast ON cell (Trace 1). Small amplitude spikes reflecting the activity of cell 1 are properly assigned by the spike sorting algorithm.
(TIF)

Figure S3 **Antidromic and orthodromic action potential propagation along the proximal axon.** (**a**) A sequence of six consecutive time frames demonstrates antidromic propagation towards the cell soma. The somatic signal is recorded by adjacent sensors in a circular region (blue sensors at time 0.36 and 0.48 msec). Red color marks +0.5 mV, blue color −0.5 mV. (**b**) Sequence of six time frames ∼2 msec after the antidromic propagation shown in (a). The signal starts near the assumed soma position and propagates orthodromic. (**c**) The sum of all sensors recording antidromic signals (not only the active sensors shown in (a)) represents the antidromic electrical footprint of the RGC. (**d**) Electrical footprint of the orthodromic spike is nearly identical to the antidromic electrical footprint shown in (c).
(TIF)
