# Supplementary figures and images for: Axonal Transmission in the Retina Introduces a Small Dispersion of Relative Timing in the Ganglion Cell Population Response
Source: PLoS One. 2011 Jun 2;6(6):e20810. doi: 10.1371/journal.pone.0020810 (PMC3107248; doi:10.1371/journal.pone.0020810)

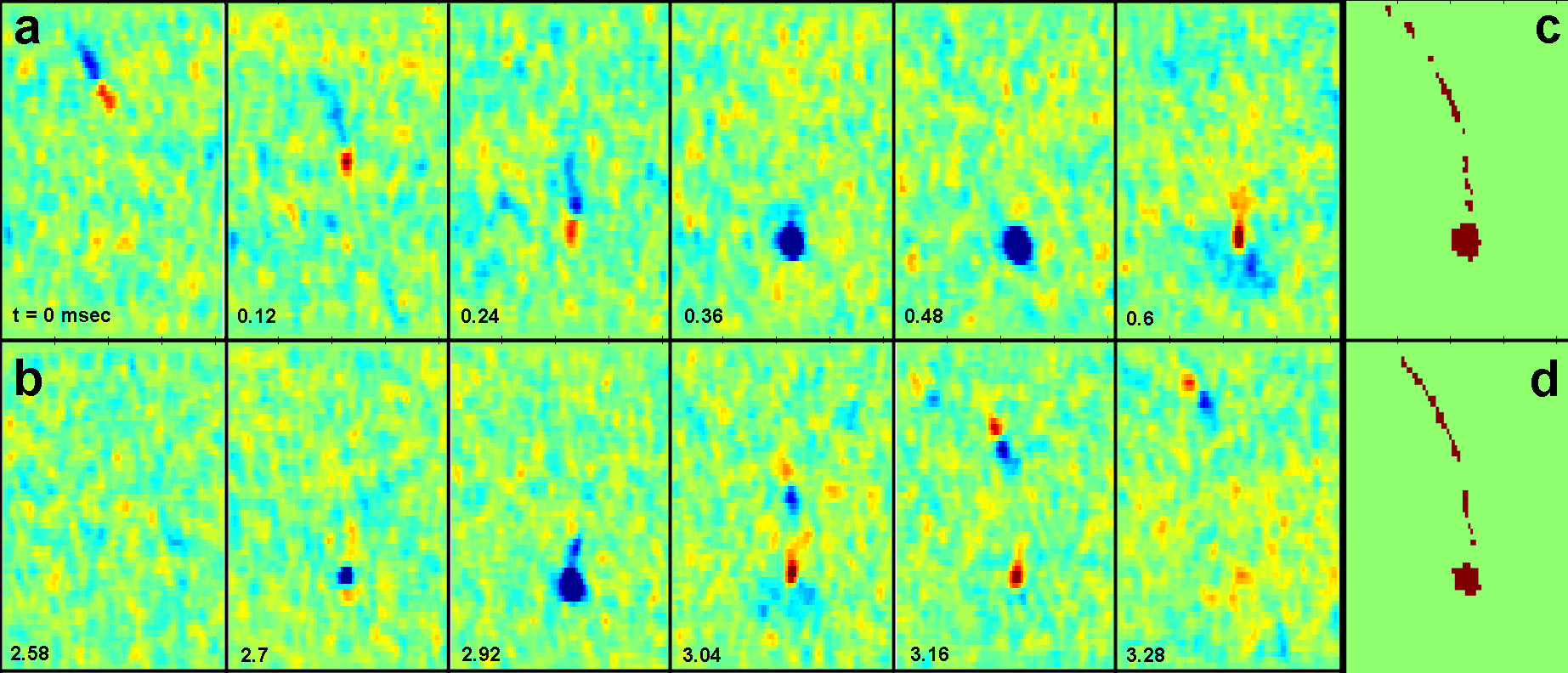

Supplement: Figure S3 — Antidromic and orthodromic action potential propagation along the proximal axon. (a) A sequence of six consecutive time frames demonstrates antidromic propagation towards the cell soma. The somatic signal is recorded by adjacent sensors in a circular region (blue sensors at time 0.36 and 0.48 msec). Red color marks +0.5 mV, blue color −0.5 mV. (b) Sequence of six time frames ∼2 msec after the antidromic propagation shown in (a). The signal starts near the assumed soma position and propagates orthodromic. (c) The sum of all sensors recording antidromic signals (not only the active sensors shown in (a)) represents the antidromic electrical footprint of the RGC. (d) Electrical footprint of the orthodromic spike is nearly identical to the antidromic electrical footprint shown in (c). (TIF) [file pone.0020810.s003.tif]
